# Vertical and Horizontal M-Charts and Microperimetry for Assessment of the Visual Function in Patients after Vitrectomy with ILM Peeling due to Stage 4 Macular Hole

**DOI:** 10.1155/2019/4975973

**Published:** 2019-05-06

**Authors:** Dominika Wrzesińska, Katarzyna Nowomiejska, Dominika Nowakowska, Agnieszka Brzozowska, Teresio Avitabile, Michele Reibaldi, Robert Rejdak, Mario Toro

**Affiliations:** ^1^Department of General Ophthalmology, Medical University of Lublin, Lublin, Poland; ^2^Department of Mathematics and Medical Biostatistics, Medical University of Lublin, Lublin, Poland; ^3^Eye Clinic, University of Catania, Catania, Italy; ^4^Department of Experimental Pharmacology, Medical Research Centre, Polish Academy of Sciences, Warsaw, Poland

## Abstract

**Purpose:**

To examine the relationship between the morphological and functional results in eyes after pars plana vitrectomy (PPV) with internal limiting membrane (ILM) peeling due to stage 4 full-thickness macular hole (FTMH).

**Methods:**

The study included 22 eyes that underwent successful PPV due to FTMH. Both vertical metamorphopsia (VM) and horizontal metamorphopsia (HM) were determined using type 2 M-charts, as well as best-corrected visual acuity (BCVA), microperimetry, and optical coherence tomography (OCT) were performed before PPV and 1 and 6 months postoperatively.

**Results:**

A significant improvement of BCVA and metamorphopsia scores measured by M-charts in particular periods before surgery, 1 and 6 months after PPV, was observed. The VM scores were consistently higher than the HM scores at all assessment times. There was a correlation found between VM and BCVA and microperimetry parameters before surgery. The macular sensitivity (MS) as well as macular integrity index increased from 1 month to 6 months after PPV and were correlated with postoperative visual acuity (VA). There was a correlation found between the hole diameter and MS and P2 parameter 6 months after PPV. There was a correlation found between mean duration of symptoms of FTMH and VA and VM score.

**Conclusions:**

VM scores seem to correlate better than HM scores with preoperative BCVA, microperimetry parameters, and duration of symptoms of the FTMH. VM scores are higher after PPV than HM scores in patients with stage 4 of the FTMH. This trial is registered with NCT03701542.

## 1. Introduction

The full-thickness macular hole (FTMH) is a defect in all layers of the neurosensorial retina including the anatomical area of the fovea with preserved intact retinal pigment epithelium [1]. FTMH affects mostly elderly people, more commonly females than males, and the female-to-male ratio is determined to be 3.3 to 1 [2]. The pars plana vitrectomy (PPV) is the most effective procedure for the treatment of FTMH, with reported closure rates of first intervention exceeding 90% [3]. The main surgical indication for vitrectomy in FTMH is stages 2, 3, and 4 [4]. The classical surgical procedure consists of the central vitreous excision, the internal limiting membrane (ILM) staining and peeling, intraocular tamponade using air or expandable gases, and postoperative patient positioning. A lot of factors have been investigated as potential predictors of final visual acuity (VA) following surgical repair of FTMH. Better surgical and functional results are associated with earlier stage of FTMH, better preoperative VA, shorter duration of symptoms, and younger patient age [5].

In clinical practice to evaluate visual function, the VA alone is usually used; however, it does not always provide a complete visual identity. The Amsler test is very simple, cheap, and consists of evenly (every 5 mm) spaced horizontal and vertical lines. The Amsler test is widely used to evaluate metamorphopsia, but it does not provide quantification of the severity of metamorphopsia [6]. M-charts (Inami, Japan) developed in 1999 by Matsumoto et al. enable evaluation of the degree of metamorphopsia quantitatively in patients with macular disease [7].

To evaluate the visual function in macular diseases, microperimetry MAIA can also be used, as it measures retinal sensitivity of the macular region, and the results can be displayed over a fundus image [8]. The microperimetry is a useful tool for objective evaluation of macular function, progression of the disease, and effectiveness of therapy [9].

The purpose of this study was to analyse metamorphopsia using the Amsler test and M-charts, as well as evaluation of macular sensitivity (MS) by using microperimetry, correlation with macular hole diameter, after successful repair of FTMH.

## 2. Methods

It was a prospective study of 22 consecutive patients who underwent successful surgery for FTMH at the Department of General Ophthalmology in Lublin, Poland. The study was performed in accordance with the Declaration of Helsinki. The approval of Ethics Committee of the Medical University of Lublin, Poland, has been given (KE-0254/269/2018). The study has been registered at ClinicalTrials.gov under the number NCT03701542. Written informed consent was obtained from all patients.

Inclusion criterion was the presentation of FTMH. Exclusion criteria were as follows: preexisting ocular diseases that could influence the outcome of the surgery, history of ocular trauma, and ocular surgery except for cataract surgery. There were 19 females and 3 males included in the study with average age of 66.73 ± 5.87 years. The surgeries were performed under local anesthesia by 2 vitreoretinal surgeons (KN and RR) using the Constellation system (Alcon, Fort Worth, Texas, US). The surgical technique characterized a standard 3-port 23, 25, or 27 gauge PPV, internal limiting membrane (ILM) peeling, fluid-air exchange, and gas tamponade (20% sulfur hexafluoride-SF6 gas). The ILM was stained in all eyes with a vital dye Brilliant Blue G (Geuder, Heidelberg, Germany). ILM was peeled off with forceps in an area of ≥2 disc diameters around the FTMH. All eyes were pseudophakic; cataract surgery was performed by phacoemulsification and implantation of foldable intraocular lens before FTMH surgery. Patients were instructed to remain in a prone position for about one week after the surgery.

Collected preoperative data were as follows: subjective duration of symptoms and diameter of the FTMH. All patients underwent best-corrected visual acuity (BCVA) and best near visual acuity (BNVA) measurements, Amsler test, M-charts, microperimetry (MAIA CenterVue, Italy), optical coherence tomography (OCT) examination OCT-2000 (Topcon Corporation, Tokyo, Japan), slit-lamp examination, and dilated funduscopy. The patients were examined before vitrectomy, 1 and 6 months after surgery.

BCVA was measured using Snellen decimal charts and converted into logMAR values. BNVA was measured using Snellen charts. The BNVA and M-charts test were performed with the best near correction, in the same lighting conditions and from the distance of 30 cm.

The type 2 M-charts consist of a double 19 dotted line with increasing dot intervals from 0.2° to 2.0° of visual angle. The test is carried out in the vertical and horizontal directions separately. The M-charts result was considered positive if the metamorphopsia score was more than 0 [6].

OCT, first introduced in 1995, became the gold standard in assessment of macular structure. OCT images were used preoperatively for the confirmation of the diagnosis and for measuring of the hole diameter and postoperatively for confirming the closure of the macular hole. Macular hole diameter was measured in micrometers as the minimum distance between the margins of FTMH. The FTMH stage was classified according to the Gass classification [10]. There was only stage 4 FTMH included. FTMH closure after surgery was determined with both a slit-lamp examination and OCT.

Microperimetry MAIA examination was performed in a dark room to analyse the retinal sensitivity and fixation stability (macular integrity index, *P*1 and *P*2 parameters). The background luminance was 4 asb, stimulus intensity range was 0–36 dB, stimulus size was Goldmann III, stimulus duration was 200 ms, and testing protocol was 4-2 threshold strategy.

Statistical analysis was performed using STATISTICA 13.0 software (StatSoft, Poland). All values were presented as the means and medians ± standard deviation, and for nonmeasurable parameters, cardinality and percentage are used. The Shapiro–Wilk test analysed measurable parameters. The Friedman ANOVA test was used to compare the preoperative and postoperative results. The R Spearman correlation was used to evaluate the relationship between the variables. *p* < 0.05 was considered to be of statistical significance.

## 3. Results

### 3.1. Visual Acuity

**Figure 1:**
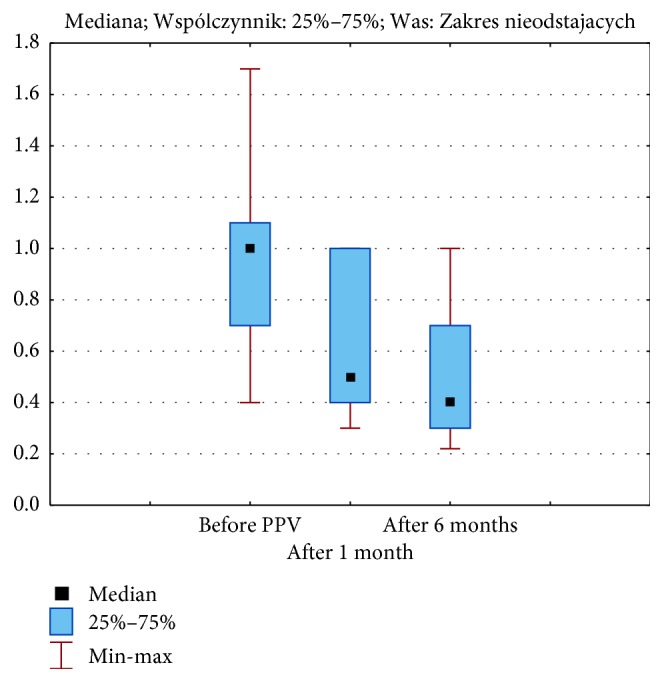
Medians, 25 and 75% quartiles, and maximum and minimum values of the visual acuity (logMAR) before pars plana vitrectomy (PPV) and after 1 and six months of the follow-up.

The mean BCVA before FTMH surgery was 1.04 ± 0.40 logMAR. The mean postoperative BCVA of all patients after 1 month was 0.64 ± 0.27 logMAR and after 6 months was 0.47 ± 0.22 logMAR (Figure 1). Statistical analysis showed a significant improvement in the BCVA after the surgery, and there were also significant differences in BCVA assessment in particular periods before surgery, at 1 and at 6 months after PPV (*p* < 0.00001).

The mean diameter of FTMH before vitrectomy was 609.45 ± 150.45 *μ*m. The mean duration of symptoms was 12.11 ± 5.90 months.

There was no correlation between BCVA before surgery and patients' age (*p*=0.28), diameter of the FTMH (*p*=1.98), and duration of symptoms (*p*=0.61).

Statistical analysis showed a significant relationship 1 month after vitrectomy between BCVA and duration of symptoms (*R* = 0.46), which was still significant after 6 months (*R* = 0.47). After 6 months, there was also a correlation between BCVA and diameter of the FTMH (*R* = 0.46).

The mean BNVA before surgery was 1.83 ± 0.68. The mean BNVA of all groups was 1.16 ± 0.47 on 1 month and 0.91 ± 0.49 on 6 months. Statistical analysis showed significant differences in BNVA before and after successful surgery (*p* < 0.00001). Preoperatively, the mean BNVA was significantly correlated with diameter of the FTMH (*R* = 0.49). It means that the higher the diameter of the FTMH, the higher the BNVA value.

There was a correlation found between BCVA before surgery and 1 and 6 months after vitrectomy. It means that better the BCVA before surgery, better the BCVA results after surgery.

### 3.2. Metamorphopsia Scores

**Figure 2:**
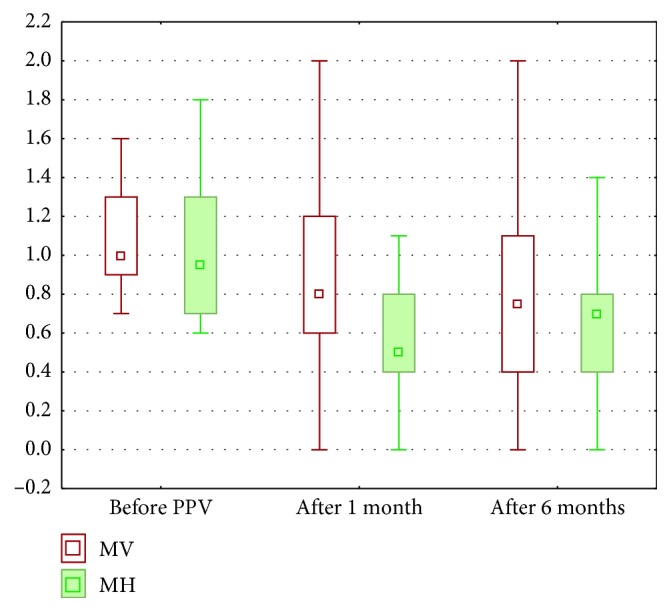
Medians, 25 and 75% quartiles, and maximum and minimum values of metamorphopsia scores both in vertical direction (VM) and horizontal direction (HM).

The mean vertical metamorphopsia (MVM) score of all patients was 1.11 ± 0.32 before surgery, improved to 0.85 ± 0.57 on 1 month and 0.86 ± 0.56 on 6 months (Figure 2). Statistical analysis showed significant differences in the VM score in particular periods before surgery, 1 and 6 months after PPV (*p*=0.002).

The mean horizontal metamorphopsia (MHM) score of all patients was 1.03 ± 0.38 before surgery, 0.64 ± 0.42 on 1 month and 0.65 ± 0.42 on 6 months. Statistical analysis showed significant differences in the HM score in particular periods before surgery, 1 and 6 months after PPV (*p*=0.00002).

The MVM scores were consistently higher than the MHM scores at all assessment times. Statistical analysis showed significant correlation between VM and HM scores, before surgery (*R* = 0.69), 1 month (*R* = 0.72), and 6 months (*R* = 0.83) after PPV.

There was a significant correlation between VM scores and microperimetry parameters before surgery, and no correlation was found between HM scores and microperimetry parameters (Table 1). No such correlation was found in regard to HM and VM scores 1 and 6 months after surgery.

Statistical analysis showed significant correlation between VM score and BCVA before vitrectomy (*R* = 0.52), but no such a correlation was found for the HM score. The higher the preoperative logMAR value, the higher the VM score. Statistical analysis did not show significant correlation between M-score (VM and HM scores) and BCVA after surgery (*p* > 0.05), as well as with preoperative diameter of FTMH at any assessment time (*p* > 0.05).

There was a correlation found between mean duration of symptoms of FTMH and VA and VM score.

### 3.3. Microperimetry

**Figure 3:**
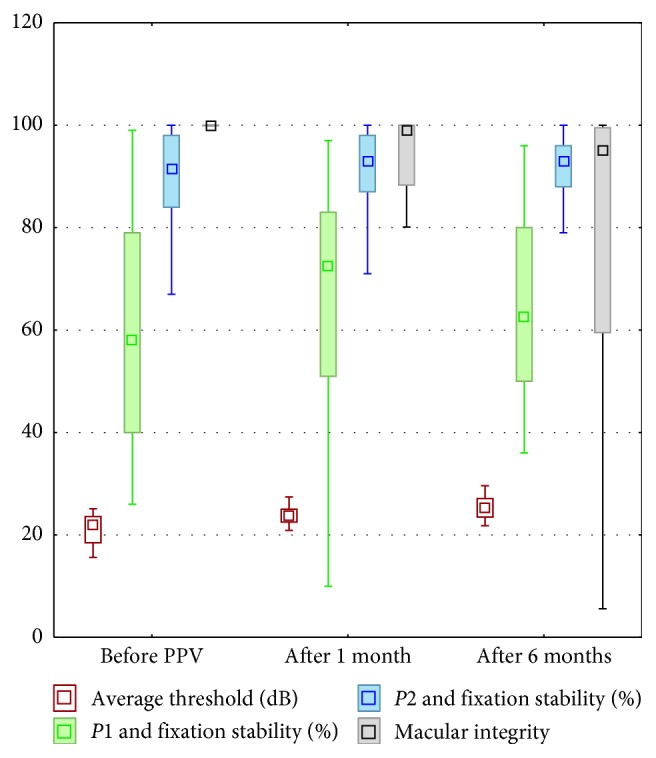
Medians, 25 and 75% quartiles, and maximum and minimum values of the following parameters in microperimetry: average threshold (in decibels), *P*1 and *P*2 fixation stability (%), and macular integrity (in decibels, dB).

Mean macular sensitivity (MS) of all patients before surgery was 21.18 ± 2.92 dB, 22.80 ± 4.37 dB after 1 month, and 24.22 ± 5.03 dB after 6 months after PPV. Statistical analysis showed significant improvement in the MS before surgery, 1 and 6 months after PPV (*p* < 0.00001) (Figure 3).

The macular integrity index was 95.12 ± 20.96 before surgery, 91.10 ± 15.77 on 1 month, and 80.80 ± 25.74 on 6 months after PPV. Statistical analysis showed significant improvement in the macular integrity index before surgery, 1 and 6 months after PPV (*p*=0.0009).

Mean fixation stability *P*1 and *P*2 values of all patients were *P*1 = 60.09 ± 24.06 and *P*2 = 88.73 ± 11.94 before surgery, *P*1 = 63.32 ± 27.05 and *P*2 = 86.05 ± 19.71 on 1 month, and *P*1 = 65.73 ± 18.56 and *P*2 = 92.00 ± 6.12 on 6 months after PPV. There were no significant differences in these parameters after statistical analysis.

Statistical analysis did not show significant correlation between microperimetry and preoperative BCVA (*p* > 0.05), while after surgery, there was a correlation between BCVA and MS (*R* = −0.64) on 1 month and on 6 months (*R* = 0.56) and between BCVA and macular integrity (*R* = 0.70) on 1 month and (*R* = −0.48) on 6 months.

There was a correlation found between FTMH diameter and MS and *P*2 parameter 6 months after PPV.

## 4. Discussion

The current study was performed to assess the functional characteristics and surgical outcomes using M-charts and microperimetry in a group of patients with FTMH.

We observed a significant improvement of BCVA and reduction of metamorphopsia after successful FTMH surgery, which was similar to the previous studies.

Kim et al. investigated the time to visual recovery after FTMH surgery. They observed significant differences in BCVA at 1 and 3 months after PPV, and BCVA stabilized after 6 months [11]. We also observed continuous improvement in VA from 1 month after PPV to 6 months after the operation. Shinoda et al. reported visual recovery at 3 months after MH surgery in a 25-gauge vitrectomy group and at 9 months after surgery in a 20-gauge vitrectomy group [12]. Mini-invasive surgery provides the shorter operation time, less trauma to the ocular surface, reduced amount of irrigation fluid, and is the reason for shorter recovery time. Directly after PPV, 77.27% of our patients had better VA results. At final follow-up period, we got the result of 100% VA improvement. It is a satisfactory outcome because Leisser et al. had BCVA improved in 83% of the cases and Amram et al. reported that 86% of eyes had improved BCVA at the final follow-up period of 1 year [13, 14]. Although percentage of patients with BCVA improvement is very important, it is necessary to estimate the level of final correction. The significant improvement of BCVA (greater than 50%) after 6 months we obtained in 50.00% of patients. The medium BCVA improvement (≥18%) was in 45.45% eyes, and 4.55% of patients had poor BCVA result (<18%). We also found the correlation between preoperative BCVA and 6 months after surgery. Better postoperative VA was correlated with better preoperative VA (*R* = 0.46), which was similar to the previous study [15].

Wendel et al. suggested better improvement in VA of patients undergoing FTMH repair with less than 6 months' duration of visual symptoms [16]. Delay from preoperative assessment to surgery can be a significant predictor of outcomes at final follow-up. In our study, duration of visual symptoms was 12.11 ± 5.90 months. Reasons for delay to surgery include patient or/and staff scheduling difficulties, lack of patient-perceived urgency, healthcare system, and other social factors.

We established lower postoperative BCVA due to larger diameter of FTMH. It is consistent with the study of Kim et al. that final visual acuity was poor in patients with a larger basal hole diameter [11].

Metamorphopsia is a common symptom affecting quality of vision due to the displacement of the photoreceptors and outer segments from their original position [17].

In our study, the Amsler test was abnormal preoperatively in 100% of patients, but it does not give the information about the severity of metamorphopsia. Ninety percent of our patients after successful surgery reported distortion of image using the Amsler test; however, the evaluation of their intensity is also difficult to assess.

In our study, the MVM score of all patients before surgery was 1.11 ± 0.32 and the MHM score was 1.03 ± 0.38. We observed a significant improvement of these parameters after surgical repair of macula. The MVM score 6 months after surgery was 0.86 ± 0.56, and the MHM score was 0.65 ± 0.42. Furthermore, the VM scores were consistently higher than the corresponding HM scores, and there was a trend—the higher the VM, the higher the HM scores at all assessment times. It is a similar result to the previous study [6, 17, 18].

In our study, the major finding was that VM scores seem to correlate better with preoperative BCVA, microperimetry parameters, and duration of symptoms of FTMH. VM scores are also higher after PPV than HM scores in patients with stage 4 FTMH. Most of the authors [7, 19, 20] present only general score of M-charts. Wada et al. analyse both HM and VM scores [6].

The higher VM score was reported and speculated that the nasal outer segments had a tendency to be thicker than other sides in FTMH, since the greater stretching of tissues toward the thicker retinal regions. The larger horizontal retinal changes may be due to the higher VM score [17].

Arimura et al. believed that the VM score was correlated with the horizontal retinal contraction and the HM score was correlated with the vertical retinal contraction [18].

Liang et al. also found that the VM score was significantly higher than the HM score, they concluded the reason behind the asymmetrically elongation, and the higher VM score remains to be further studied. Liang et al. also reported the relationship between M-score and macular parameters. They found that the postoperative mean M-score was significantly positively correlated with the mean minimal diameter of FTMH, but there was no correlation between preoperative M-score and the mean minimal diameter. Furthermore, they proposed that the mean minimal diameter of FTMH may be a prognostic factor for postoperative metamorphopsia, which may help one to predict postoperative metamorphopsia before surgery [17]. Our results contrast with this study, because we did not find any correlation between M-scores and minimal diameter of FTMH. Also, the other researchers showed no significant correlations between the VM and HM scores and any of the preoperative OCT parameters at any assessment time [6, 19]. It may reflect a difference in the average minimum diameter of the FTMH in different study populations. In addition, in our study, there was a significant correlation between preoperative VM score and BCVA. We agree with the other researchers that preoperative metamorphopsia measured by M-charts may have more complicated affected factors besides macular parameters and evaluating M-scores, especially VM score in addition to BCVA can be an independent treatment outcome [6, 17].

To assess the functional success of the surgery, we used microperimetry measuring retinal sensitivity and the fixation behavior while fundus is directly examined. It is rapid, safe, noninvasive, and useful tool for evaluation of macular function [9]. We observed preoperatively a significant loss of sensitivity of macular area affected by a disease, and the mean sensitivity (dB) was 21.18 ± 2.92. Six months after surgery, the mean sensitivity was better (24.22 ± 5.03). The macular integrity index is a numerical value that describes the likelihood that a patient's responses are normal, suspect, or abnormal when compared to age-adjusted normative data. It does not represent the severity of the disease process. There is no direct relationship between the average threshold value (dB) and the macular integrity index. Preoperatively, in our group, the mean macular integrity index was 95.12 ± 20.96 and 6 months after surgery was 80.80 ± 25.74. The lower value of the macular integrity index means the better respond and postoperative improvement of this parameter. We found the correlation between the postoperative mean MS and the preoperative size of FTMH. The larger diameter of FTMH provides the lower value of MS. The higher MS and lower macular integrity index are correlated with better postoperative BCVA. Our results and connections between MS and diameter of FTMH or MS and quality of postoperative BCVA are similar to the result previously shown by Bonnabel et al. [15]. It needs to be highlighted that, we did not find any significant correlation between MS and preoperative BCVA (*p* > 0.05), so microperimetry can be an additional, useful tool in predicting the postoperative prognosis and objective evaluation of changes in visual outcome. Additionally, we found correlation between microperimetry and M-score before PPV. Parameters such as average threshold value, *P*1, and *P*2, were correlated with the VM score. There were no similar connections with the HM score (*p* > 0.05). The lower VM score provides the better MS, *P*1, and *P*2 results.

Preoperative, intraoperative, and postoperative factors have been investigated as potential predictors of final VA following surgical repair of FTMH. Among preoperative factors, two well established are symptom duration and preoperative VA. The other factors are patient age and OCT parameters such as stage of FTMH, minimum diameter or/and base diameter of the FTMH, central subfield retinal thickness, height of the FTMH and diameter of internal/external segments layer IS/OS, and external limiting membrane (ELM) defects [5, 21, 22]. However, these parameters are anatomical, imaging-based features, and to estimate the result of FTMH surgery, functional tests are required too.

In summary, this study evaluated prospectively metamorphopsia using M-charts and Amsler test, and MS result in microperimetry MAIA among patients after successful vitrectomy due to FTMH. Both microperimetry and M-charts test, especially VM-score, provide more comprehensive information of the visual function of FTMH patients before and after surgery and can be useful, additional predictor factors of succeed vitrectomy and postoperative results.

## Figures and Tables

**Table 1:** Correlation between horizontal (HM) and vertical (VM) M-charts and microperimetry parameters.

Parameters	VM		HM	
	*R*	*p*	*R*	*p*
Average threshold (dB)	−0.47	0.03^*∗*^	−0.39	0.08
*P*1	−0.51	0.02^*∗*^	−0.38	0.08
*P*2	−0.48	0.02^*∗*^	−0.35	0.11
Macular integrity	−0.02	0.92	0.03	0.90

^*∗*^ *p* < 0.05 indicates that significant correlations were found between two parameters.

## Data Availability

The data used to support the findings of this study are available from the corresponding author upon request.

## References

[1] Kumari K., Tahir M. A., Cheema A. (2017). Visual and anatomical outcome of macular hole surgery at a tertiary healthcare facility. *Pakistan Journal of Medical Sciences*.

[2] McCannel C. A., Ensminger J. L., Diehl N. N., Hodge D. N. (2009). Population-based incidence of macular holes. *Ophthalmology*.

[3] Kusuhara S., Ooto S., Kimura D. (2008). Outcomes of 23- and 25-gauge transconjunctival sutureless vitrectomies for idiopathic macular holes. *British Journal of Ophthalmology*.

[4] Merticariu A., Balta F., Pop M. (2015). Macular hole treatment. *Journal of Translational Medicine and Research*.

[5] Jenisch T. M., Zeman F., Koller M., Märker D. A., Helbig H., Herrmann W. A. (2017). Macular hole surgery: an analysis of risk factors for the anatomical and functional outcomes with a special emphasis on the experience of the surgeon. *Clinical Ophthalmology*.

[6] Wada I., Yoshida S., Kobayashi Y. (2017). Quantifying metamorphopsia with M-CHARTS in patients with idiopathic macular hole. *Clinical Ophthalmology*.

[7] Matsumoto C., Arimura E., Okuyama S., Takada S., Hashimoto S., Shimomura Y. (2003). Quantification of metamorphopsia in patients with epiretinal membranes. *Investigative Opthalmology & Visual Science*.

[8] Nizawa T., Baba T., Kitahashi M., Oshitari T., Yamamoto S. (2017). Different fixation targets affect retinal sensitivity obtained by microperimetry in normal individuals. *Clinical Ophthalmology*.

[9] Laishram M., Srikanth K., Rajalakshmi A. R., Nagarajan S., Ezhumalai G. (2017). Microperimetry—a new tool for assessing retinal sensitivity in macular diseases. *Journal of Clinical and Diagnostic Research*.

[10] Gass J. D. M. (1987). *Stereoscopic Atlas of Macular Diseases: Diagnosis and Treatment*.

[11] Kim S. H., Kim H. K., Yang J. Y., Lee S. C., Kim S. S. (2018). Visual recovery after macular hole surgery and related prognostic factors. *Korean Journal of Ophthalmology*.

[12] Shinoda H., Shinoda K., Satofuka S. (2008). Visual recovery after vitrectomy for macular hole using 25-gauge instruments. *Acta Ophthalmologica*.

[13] Leisser C., Palkovits S., Hirnschall N. (2018). One-year results after internal limiting membrane flap transposition for surgical repair of macular holes with respect to microperimetry. *Ophthalmic Research*.

[14] Amram A. L., Mandviwala M. M., Ou W. C., Wykoff C. C., Shah A. R. (2018). Predictors of visual acuity outcomes following vitrectomy for idiopathic macular hole. *Ophthalmic Surgery, Lasers and Imaging Retina*.

[15] Bonnabel A., Bron A. M., Isaico R., Dugas B., Nicot F., Creuzot-Garcher C. (2013). Long-term anatomical and functional outcomes of idiopathic macular hole surgery: the yield of spectral-domain OCT combined with microperimetry. *Graefe’s Archive for Clinical and Experimental Ophthalmology*.

[16] Wendel R. T., Patel A. C., Kelly N. E., Salzano T. C., Wells J. W., Novack G. D. (1993). Vitreous surgery for macular holes. *Ophthalmology*.

[17] Liang X., Wang Y., Liu L. (2018). Relationship between metamorphopsia and macular parameters before and after idiopathic macular hole surgery. *Ophthalmic Surgery, Lasers and Imaging Retina*.

[18] Arimura E., Matsumoto C., Okuyama S., Takada S., Hashimoto S., Shimomura Y. (2005). Retinal contraction and metamorphopsia scores in eyes with idiopathic epiretinal membrane. *Investigative Opthalmology & Visual Science*.

[19] Krasnicki P., Dmuchowska D. A., Pawluczuk B., Proniewska-Skretek E., Mariak Z. (2015). Metamorphopsia before and after full-thickness macular hole surgery. *Advances in Medical Sciences*.

[20] Uei B., Lee Z., Shimada H., Yuzawa M. (2005). Preoperative factors for postoperative resolution of metamorphopsia in idiopathic macular hole surgery. *Nihon Ganka Gakkai Zasshi*.

[21] Karatepe A. S., Menteş J., Erakgün E. T. (2018). Vitreoretinal interface characteristics in eyes with idiopathic macular holes: qualitative and quantitative analysis. *Turkish Journal of Ophthalmology*.

[22] Shpak A. A., Shkvorchenko D. O., Sharafetdinov I. K., Yukhanova O. A. (2016). Predicting anatomical results of surgical treatment of idiopathic macular hole. *International Journal of Ophthalmology*.

